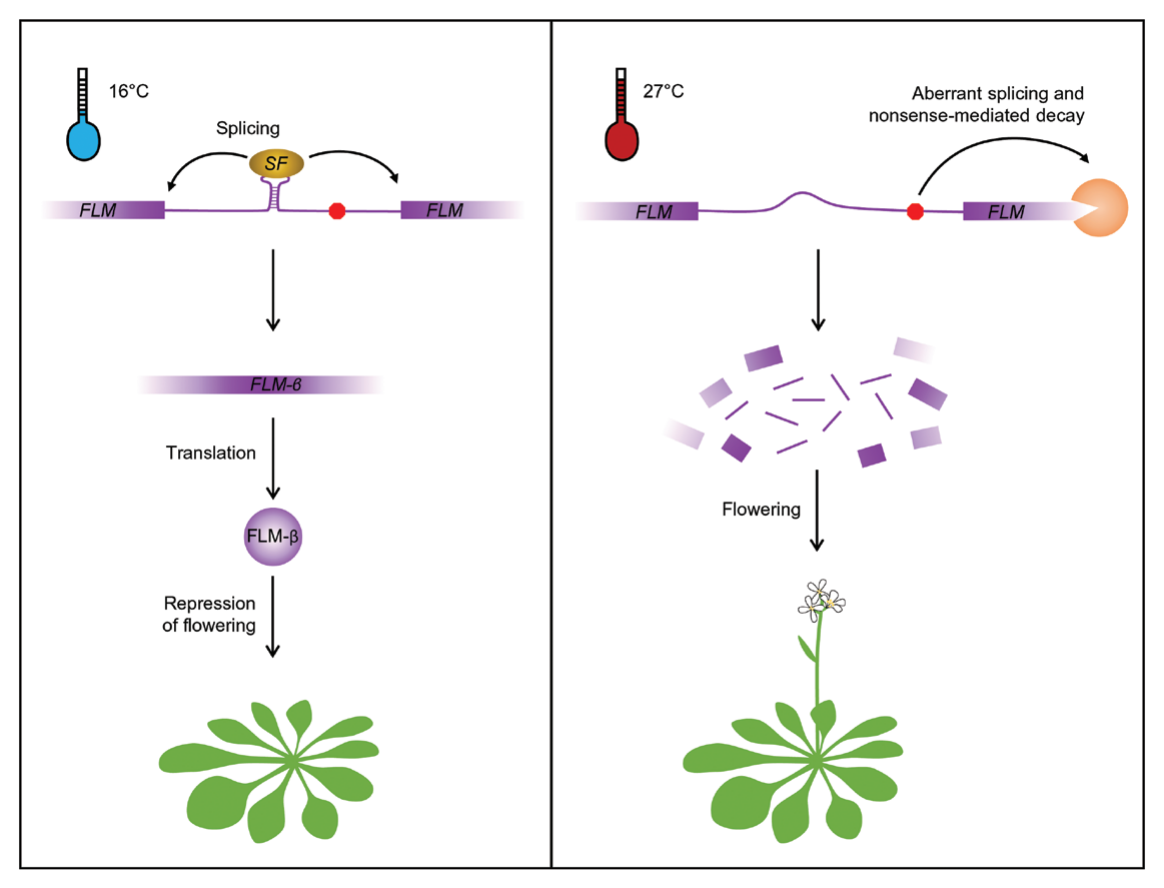# Regulation of flowering time: a splicy business

**DOI:** 10.1093/jxb/erx353

**Published:** 2017-11-01

**Authors:** Rainer Melzer

**Affiliations:** School of Biology and Environmental Science, University College Dublin, Dublin, Ireland

**Keywords:** Alternative splicing, Arabidopsis, *FLM* isoforms, FLOWERING LOCUS M (FLM), flowering time, temperature-dependent mediated flowering

## Abstract

This article comments on:

**Capovilla G, Symeonidi E, Wu R, Schmid M.** 2017. Contribution of major *FLM* isoforms to temperature-dependent mediated flowering in *Arabidopsis thaliana*. Journal of Experimental Botany **68,** 5117–5127.

**The mechanisms regulating flowering time at ambient temperatures are controversial. One hypothesis ascribes prominent roles to two competing protein isoforms of FLOWERING LOCUS M (FLM) which are produced by alternative splicing. Now, Capovilla *et al.* (2017) have demonstrated that alternative splicing of *FLM* is indeed important, but that competing protein isoforms are probably of much lesser relevance than originally thought.**

Few decisions in life are more important than getting the time for reproduction right. If a bad choice is made one may not be able to find a suitable partner, conditions for producing progeny might be suboptimal or the progeny might be forced to grow up in an unsuitable environment. To prevent that, elaborate mechanisms have evolved in many species. In plants, the switch from vegetative to reproductive development is controlled by dozens of genes integrated in numerous well known pathways (Srikanth and Schmid, 2011). However, it was discovered only relatively recently that ambient temperature plays a critical role in controlling flowering time: many Arabidopsis ecotypes flower substantially earlier at 27°C than at the more usual growth temperature of 23°C (Balasubramanian *et al.*, 2006). This ambient temperature pathway has been the subject of intense research over the past few years, not least because in times of global warming the analysis of genetic circuits responding to temperature changes has gained considerable importance.

One of the key genes identified was *FLOWERING LOCUS M*, which encodes a MADS-domain transcription factor (Scortecci *et al.*, 2001; Balasubramanian *et al.*, 2006). In Arabidopsis plants in which *FLM* is deleted, early flowering is observed no matter whether the plants are grown at 23°C or 27°C (Balasubramanian *et al.*, 2006). This indicates that *FLM* is a repressor of early flowering at lower temperatures (Balasubramanian *et al.*, 2006).

To exert its repressive effect, FLM interacts with another MADS-domain protein, SHORT VEGETATIVE PHASE (SVP) (Lee *et al.*, 2013). Like FLM, SVP is also involved in regulating flowering time at ambient temperatures (Lee *et al.*, 2007). The current view is that SVP/SVP homomeric and SVP/FLM heteromeric protein complexes delay flowering at lower temperatures by repressing genes that act as floral activators (Lee *et al.*, 2013; Pose *et al.*, 2013). But exactly how is this repression released at higher temperatures? In the case of SVP, one hypothesis is that the protein shows a marked decrease in stability at higher temperatures (Lee *et al.*, 2013). *FLM*, on the other hand, is subject to alternative splicing, and the occurrence of specific splice forms is correlated to the growth temperature (Balasubramanian *et al.*, 2006; Pose *et al.*, 2013). The two major splice forms of *FLM* are designated *FLM-β* and *FLM-δ* (Scortecci *et al.*, 2001). *FLM-β* is the predominant transcript at lower temperatures, whereas *FLM-δ* appears to be more enriched at higher temperatures (Pose *et al.*, 2013). The FLM-β and FLM-δ proteins are capable of interacting with SVP. However, only FLM-β and not FLM-δ forms DNA-binding complexes with SVP (Pose *et al.*, 2013). In addition, overexpression of FLM-β resulted in late flowering, whereas overexpression of FLM-δ led to earlier flowering in comparison to wild-type plants (Pose *et al.*, 2013).

This gave rise to an appealing hypothesis: the FLM-β and FLM-δ proteins compete with each other in binding to SVP. However, only the FLM-β/SVP complex is capable of repressing flowering, because FLM-δ/SVP lacks DNA-binding activity. At lower temperatures, FLM-β/SVP and SVP/SVP complexes predominate and hence flowering is repressed. In contrast, with an increase in temperature, FLM-δ becomes more abundant, outcompetes FLM-β and SVP itself for SVP binding and hence flowering is activated (Pose *et al.*, 2013).

## FLM-δ under scrutiny

However, as exciting as the hypothesis of a dominant-negative protein variant generated by alternative splicing is (especially for those researchers with a strong interest in the biophysics of transcription factor functions), it has been challenged in recent years.

For example, although *FLM-β* transcripts decrease with increased temperatures, a concomitant increase in *FLM-δ* cannot always be detected (Sureshkumar *et al.*, 2016). Those and other findings led to the competing hypothesis that the decrease in *FLM-β* transcripts (and with this FLM-β protein) is the main determinant of early flowering at higher temperatures and that the contribution of the FLM-δ protein is negligible (Lutz *et al.*, 2015; Sureshkumar *et al.*, 2016; Lutz *et al.*, 2017).

Capovilla *et al.* (2017) take a fresh look at the role of FLM-δ in flowering-time regulation. Towards that goal, they produced Arabidopsis lines that are incapable of expressing either *FLM-β* or *FLM-δ* . To circumvent potential problems and artefacts associated with the expression of transgenic *FLM* versions, the genomic *FLM* locus was edited using CRISPR-Cas9, creating plants that lacked either the 2nd or 3rd exon of *FLM*. As the 2nd exon is part of the *FLM-β* but not of the *FLM-δ* transcript, plants lacking it can produce *FLM-δ* but not *FLM-β* . Conversely, plants lacking the 3rd exon are incapable of producing *FLM-δ* but can produce *FLM-β* . This set-up makes it possible to more clearly distinguish whether the *FLM-δ* transcript (and protein) plays an important role in controlling flowering time: if the FLM-δ protein is a dominant-negative version that competes with FLM-β for binding to SVP (Pose *et al.*, 2013), plants expressing only FLM-δ might be expected to flower earlier than *flm* loss-of-function mutants. This is because in those plants FLM-δ would prevent SVP homodimer formation (something that wouldn’t be possible in *flm* loss-of-function mutants) and hence flowering can proceed. This, however, was not observed. The ‘FLM-δ only’ plants flowered as early as full loss-of-function mutants, indicating that there is no substantial dominant-negative effect exerted by the FLM-δ protein. Thus, the results clearly favour the idea that decreasing levels of the *FLM-β* transcripts alone trigger flowering at elevated temperatures (Capovilla *et al.*, 2017).

## Splicing and the quest for the temperature signalling pathway in plants

One surprising result from the study of Capovilla *et al.* (2017) was that plants only capable of producing *FLM-β* and not *FLM*- *δ* flowered later than wild-type plants. This is initially not easy to reconcile with the idea that *FLM-δ* is a non-functional transcript. After all, if *FLM-δ* has no function, why should it matter whether the plant is capable of producing it? Transcript analysis provides an important clue here: the results of Capovilla *et al.* (2017) and others (Sureshkumar *et al.*, 2016) indicate that the temperature-dependent variation in *FLM-β* levels is not achieved by different rates of transcription but by alternative splicing. At higher temperatures, splicing produces less *FLM-β* and more alternative transcripts, among them *FLM-δ* but also others, many of which contain premature stop codons and may be subjected to nonsense-mediated decay (Sureshkumar *et al.*, 2016). Intriguingly, one of the major temperature-sensitive splice donor sites that is predominantly used at higher temperatures is that of intron 3, and exactly that site was removed in the ‘*FLM-β* only’ plants (Sureshkumar *et al.*, 2016; Capovilla *et al.*, 2017). So, what presumably happens in those plants is that more *FLM-β* is produced because the alternative splice site for producing *FLM-δ* and other splice variants is missing. And more *FLM-β* leads to delayed flowering.

This highlights an important corollary of alternative splicing: the production of alternative, non-functional transcripts can be an important regulatory mechanism to reduce the amount of functional transcripts (Reddy *et al.*, 2013). It is becoming increasingly clear that transcript abundance of many genes is regulated by this ‘alternative splicing nonsense-mediated decay’ pathway (Reddy *et al.*, 2013).

Interestingly, many of the RNA-binding proteins implicated in alternative splicing are subject to alternative splicing themselves, and this splicing can also be susceptible to temperature variations (Lazar and Goodman, 2000; Duque, 2011; Capovilla *et al.*, 2015). It is therefore tempting to speculate that the temperature-dependent variation in abundance and/or activity of splicing factors is responsible for temperature-dependent splicing of *FLM* (Box 1). An ‘alternative splicing–alternative splicing’ cascade may exist in which *FLM* utilizes a pre-existing circuit that is susceptible to temperature variations (Verhage *et al.*, 2017). Importantly, it was recently discovered that Phytochromes act as temperature sensors in plants and that they possess RNA-binding activity (Jung *et al.*, 2016; Legris *et al.*, 2016; Reichel *et al.*, 2016). One may therefore speculate that Phytochromes act as splicing factors that are involved in the temperature-dependent regulation of *FLM* (Köster *et al.*, 2017).

In general, the secondary structure of RNA is also highly susceptible to temperature changes and an important factor that determines splicing patterns (Reddy *et al.*, 2013; Vandivier *et al.*, 2016). It does therefore also appear possible that the structure of the *FLM* pre-mRNA or the pre-mRNA structure of a splice factor acts as a sensor in the ambient temperature pathway (Box 1).

This would also explain why a more ‘common’ developmental mechanisms of transcript abundance regulation, like a temperature-dependent rate of transcription, is not observed for *FLM*: if the RNA of *FLM* or a splice factor is a temperature sensor, *FLM* has to be expressed to sense the temperature.

## Rise and fall of a hypothesis

The achievements in unravelling the molecular mechanism of *FLM* function notwithstanding, the study by Capovilla *et al.* (2017) might be even more important from the perspective of how science is conducted. The original idea of FLM-δ acting as a dominant-negative protein version made perfect sense at the time it was published (Pose *et al.*, 2013). Indeed, it soon became a standard example on the importance of protein isoforms produced by alternative splicing in plants (Staiger and Brown, 2013; Pajoro *et al.*, 2014). But subsequent papers didn’t find evidence for an important role of the FLM-δ protein (Lutz *et al.*, 2015; Sureshkumar *et al.*, 2016; Lutz *et al.*, 2017). So, the same group that developed the original hypothesis was also involved in studies contradicting it (Lutz *et al.*, 2015), and with Capovilla *et al.* (2017) they themselves provided the last nail in the coffin of the FLM-δ hypothesis. This is the essence of how science should be conducted: formulating a hypothesis, testing it rigorously and, if necessary, rejecting it. However, we all know that this is not always easy. As Max Planck once put it (Planck, 1949): ‘A new scientific truth does not triumph by convincing its opponents and making them see the light, but rather because its opponents eventually die, and a new generation grows up that is familiar with it.’ Capovilla *et al.* (2017) prove that this is not always the case and that scientists can (and maybe more often should) change their mind in the light of new evidence.

**Box 1. Hypothetical scenario as to how *FLM* controls flowering time in response to temperature differences**

At lower temperatures (left), the *FLM* pre-mRNA is correctly spliced, yielding the *FLM-β* transcript. The FLM-β protein represses flowering. At higher temperatures (right), splicing is disturbed, triggering nonsense-mediated decay through premature stop codons (red octagon). Consequently, less (or no) FLM-β protein is produced and flowering is activated. *FLM-δ* is very likely also produced, but is not shown here as the protein presumably has no role in flowering-time regulation.

The presence/absence of splicing factors (SF) may contribute to alternative splicing. It is not yet entirely clear how plants sense temperature differences, but one possibility is that temperature-dependent RNA structures (symbolized by the presence/absence of the stem-loop structure) are involved.

**Figure:**